# Functionally Related Genes Cluster into Genomic Regions That Coordinate Transcription at a Distance in Saccharomyces cerevisiae

**DOI:** 10.1128/mSphere.00063-19

**Published:** 2019-03-13

**Authors:** Alanna Cera, Maria K. Holganza, Ahmad Abu Hardan, Irvin Gamarra, Reem S. Eldabagh, Megan Deschaine, Sarah Elkamhawy, Exequiel M. Sisso, Jonathan J. Foley, James T. Arnone

**Affiliations:** a Department of Biology, William Paterson University, Wayne, New Jersey, USA; b Department of Chemistry, William Paterson University, Wayne, New Jersey, USA; Carnegie Mellon University

**Keywords:** coregulation, genomics, *Saccharomyces cerevisiae*, gene expression

## Abstract

Balancing gene expression is a fundamental challenge of all cell types. To properly regulate transcription on a genome-wide level, there are myriad mechanisms employed by the cell. One layer to this regulation is through spatial positioning, with particular chromosomal loci exerting an influence on transcription throughout a region. Many coregulated gene families utilize spatial positioning to coordinate transcription, with functionally related genes clustering together which can allow coordinated expression via adjacent gene coregulation. The mechanisms underlying this process have not been elucidated, though there are many coregulated gene families that exhibit this genomic distribution. In the present study, we tested for a role for the enhancer-promoter (EP) hypothesis, which demonstrates that regulatory elements can exert transcriptional effects over a broad distance, in coordinating transcriptional coregulation using budding yeast, Saccharomyces cerevisiae. We empirically validated the EP model, finding that the genomic distance a promoter can affect varies by locus, which can profoundly affect levels of transcription, phenotype, and the extent of transcriptional disruption throughout a genomic region. Using the nitrogen metabolism, ribosomal protein, toxin response, and heat shock gene families as our test case, we report functionally clustered genes localize to genomic loci that are more conducive to transcriptional regulation at a distance compared to the unpaired members of the same families. Furthermore, we report that the coregulation of functional clusters is dependent, in part, on chromatin maintenance and remodeling, providing one mechanism underlying adjacent gene coregulation.

**IMPORTANCE** The two-dimensional, physical positioning of genes along a chromosome can impact proper transcriptional regulation throughout a genomic region. The transcription of neighboring genes is correlated in a genome-wide manner, which is a characteristic of eukaryotes. Many coregulated gene families can be found clustered with another member of the same set—which can result in adjacent gene coregulation of the pair. Due to the myriad gene families that exhibit a nonrandom genomic distribution, there are likely multiple mechanisms working in concert to properly regulate transcriptional coordination of functionally clustered genes. In this study, we utilized budding yeast in an attempt to elucidate mechanisms that underlie this coregulation: testing and empirically validating the enhancer-promoter hypothesis in this species and reporting that functionally related genes cluster to genomic regions that are more conducive to transcriptional regulation at a distance. These clusters rely, in part, on chromatin maintenance and remodelers to maintain proper transcriptional coordination. Our work provides insight into the mechanisms underlying adjacent gene coregulation.

## Abstract (precis)

The two-dimensional, physical positioning of genes along a chromosome can impact proper transcriptional regulation throughout a genomic region. The transcription of neighboring genes is correlated in a genome-wide manner, which is a characteristic of eukaryotes. Many coregulated gene families can be found clustered with another member of the same set—which can result in adjacent gene coregulation of the pair. Due to the myriad gene families that exhibit a nonrandom genomic distribution, there are likely multiple mechanisms working in concert to properly regulate transcriptional coordination of functionally clustered genes. In this study, we utilized budding yeast in an attempt to elucidate mechanisms that underlie this coregulation: testing and empirically validating the enhancer-promoter hypothesis in this species and reporting that functionally related genes cluster to genomic regions that are more conducive to transcriptional regulation at a distance. These clusters rely, in part, on chromatin maintenance and remodelers to maintain proper transcriptional coordination. Our work provides insight into the mechanisms underlying adjacent gene coregulation.

## INTRODUCTION

Proper regulation of transcription is essential for cellular survival through the establishment and maintenance of homeostasis within a particular environmental and developmental context. To balance transcription on a genome-wide scale, myriad layers of regulation have evolved and are utilized, including *cis* regulatory DNA sequences, such as promoters and enhancers, that recruit *trans*-acting, DNA-binding transcription factors (1–3). Transcription factors work in either singular or combinatorial fashion to recruit RNA polymerases, ultimately altering the rate of production of RNA to meet cellular demands (4, 5). The accessibility of *cis* regulatory sequences, and the corresponding transcription factors, is altered by the formation of differential chromatin structures resulting from changes in the location, modification, and composition of histones and nucleosome complexes (6–8).

Spatial positioning, the two-dimensional arrangement of genes along the chromosome, can profoundly influence transcription throughout a genomic region. In eukaryotes, genes positioned adjacent to heterochromatin ultimately are converted to heterochromatin and are silenced by either the telomere proximal effect (TPE) or by position effect variegation (PEV) (9–11). Position effects are not limited to gene repression; localized clusters of genes are transcribed and transcriptionally correlated throughout the genome in many eukaryotic organisms, including the Saccharomyces cerevisiae, Drosophila melanogaster, Arabidopsis thaliana, and Danio rerio genomes, and this is conserved up through humans (Homo sapiens) (12–15). This transcriptional correlation is partially mediated by enhancer and promoter promiscuity, termed the enhancer-promoter (EP) theory, whereby the activity of regulatory elements extends over a broad distance to regulate multiple targets scaling in accordance to genome size (16).

One regulatory challenge involves the coordinated expression of functionally related gene families whose composite members are required in stoichiometric levels by the cell. The best characterized examples of this are represented in the coregulated gene families, or regulons, that are required for production of the ribosome. In eukaryotic organisms, each ribosome requires the coordinated production of four highly modified and folded rRNAs, approximately 80 ribosomal proteins (RPs), and more than 200 rRNA and ribosomal biogenesis (RRB) processing and assembly factors (17). Central to translation, and intimately connected to cell growth, the ribosome is tightly regulated in eukaryotes (18). Numerous studies have unraveled many layers coordinating transcription of these components, including the *cis* and *trans* factors specific to the RP and RRB regulons (19, 20). One interesting observation revealed that the RP and RRB genes exhibit a nonrandom, statistically significant distribution throughout the genome; the RP and RRB genes are found clustered with other members of the same regulon (but not with members across the regulons). This genomic distribution is conserved throughout divergent fungal lineages and more complex eukaryotic organisms (21, 22). The members of both the RP and RRB families are predominantly found as pairs. In S. cerevisiae, spatial proximity allows for adjacent gene coregulation, whereby the clustered genes are coregulated via shared regulatory elements functioning at a distance (23, 24). Many functionally related gene families exhibit this nonrandom distribution of their members throughout the genome as functional clusters. This characteristic is conserved in broadly divergent eukaryotes and coordinates transcriptional coregulation of these gene families (25).

In this current work, we use the budding yeast, Saccharomyces cerevisiae, to empirically test the EP theory as a mechanism underlying adjacent gene coregulation. Our findings reveal that the distance a promoter can activate a gene varies based on both its proximity to a gene and the site of integration. This manifests at the level of transcriptional activation, in changes in growth phenotype, and in the degree of disruption surrounding the integration locus. We report that adjacent gene coregulation is mediated, in part, by the clustering of functionally related genes into genomic regions that are more conducive to transcriptional regulation at a distance compared to their unpaired counterparts. Furthermore, we identify a putative role for chromatin remodeling in the coregulation of functional clusters and identify several chromatin remodeling complexes that are necessary.

## RESULTS

### The level of expression induced by a UAS_Gal_–*HIS3* reporter decays at a rate that varies depending on the site of integration.

**FIG 1:**
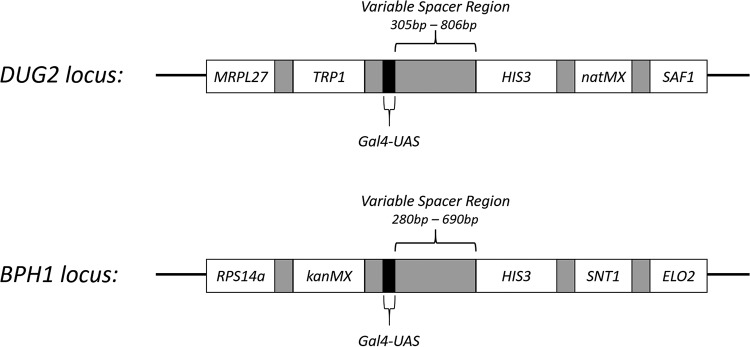
Schematic showing the reporter construct at the sites of integration in the strains utilized in this study. The location and spatial arrangement of the reporter constructs at both the *DUG2* locus (top) and the *BPH1* (bottom) locus. The *HIS3* gene was separated from the UAS_Gal_ by a variable length spacer in each strain. See Table 4 for the complete relevant genotype for every strain used in this study.

**FIG 2:**
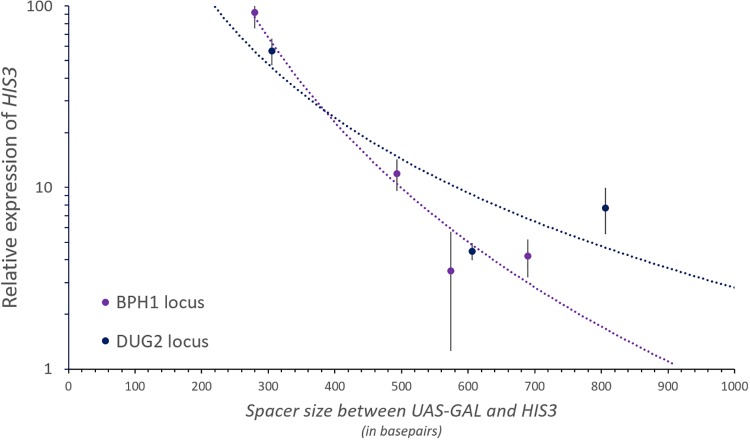
Relative levels of *HIS3* gene expression and activation at the *BPH1* and *DUG2* loci. Expression of *HIS3* was determined relative to *ACT1* and plotted as a function of the size of the spacer separating the distance of UAS_Gal_ from the gene. Points on the plot represent the average level of expression, and error bars depict the standard errors of the means. The decay curves are color coded by locus and extrapolated to estimate the *x*-axis intercepts.

To test the EP theory, we began by taking advantage of a series of yeast strains that have been previously developed, where an inducible *HIS3* reporter gene was placed under the influence of a galactose-inducible upstream activating sequence (UAS_Gal_) to monitor the effects of transcriptional activation at a distance as a function of genomic location (Fig. 1) (26). The spacing between the *HIS3* gene and the UAS_Gal_ varied between 280 and 806 bp across a series of yeast strains with the construct integrated into either the *BPH1* locus on chromosome III or into the *DUG2* locus found on chromosome II (26). We began by utilizing quantitative reverse transcription-PCR (qRT-PCR) to measure the relative expression of *HIS3* upon the activation of transcription by the addition of galactose to the media. The level of *HIS3* expression was plotted as a function of spacer size separating the two elements (Fig. 2). When the spacer between *HIS3* and UAS_Gal_ was smallest (approximately 300 bp), the level of *HIS3* expression was roughly comparable between the two sites of integration. As the size of the spacer increased, a concomitant decrease in the level of *HIS3* expression was observed at each locus. However, the rate of this decrease varied at each site. The rate of decay at the *BPH1* locus was quicker than the rate of decay seen at the *DUG2* locus, and we can extrapolate a complete loss of *HIS3* activation predicted to occur after approximately 920 bp versus 1,500 bp, respectively.

**FIG 3:**
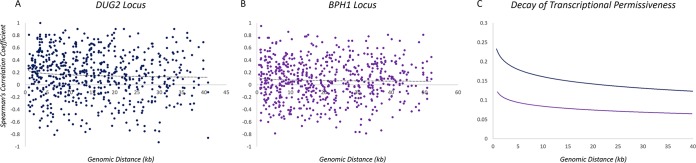
Extent of transcriptional coregulation across the genomic neighborhood surrounding the site of reporter integration. (A and B) Spearman’s correlation coefficient was calculated for every pairwise combination within the 10-gene neighborhood surrounding the *DUG2* locus (A) and the *BPH1* locus (B). (C) The decay curves determined for each locus were color coded and are plotted together for comparison of the permissiveness.

As the difference in the rate of decay observed at the *BPH1* locus and the *DUG2* locus was larger than we initially expected, we characterized the extent of coregulated expression throughout each locus. Using a bioinformatic approach, we calculated the Spearman’s correlation coefficient (SCC) throughout the 10-gene window surrounding each site of integration. Gene expression profiles were extracted from the following microarray data sets: cycling cells, a heat shock time course, a DNA damage response time course, an oxidative stress time course, a nitrogen depletion time course, carbon source switching, and nutrient limitation from carbon, nitrogen, phosphorus, or sulfur (27–29). The SCC was calculated for every pairwise gene combination within the window and plotted as a function of genomic distance (Fig. 3).

Within both the *BPH1* locus and the *DUG2* locus, both positive correlations and negative correlations (anticorrelations) are observed between specific genes and across different expression conditions (Fig. 3A and B). The transcriptional decay curve was calculated for each locus to determine the extent of coregulated expression throughout each region (Fig. 3A and B). The decay calculations represent the average absolute value of the SCCs. For clarity, the decay curves were overlaid for comparison between each locus as a separate plot (Fig. 3C). Both loci exhibit a positive correlation that decays as a function of distance throughout each window, consistent with previous reports (16). The correlation is higher and decays at a lower rate at the *DUG2* locus (*y* = −0.027ln *x* + 0.2233; *R*^2^ = 0.0044) than at the *BPH1* locus (*y* = −0.013ln *x* + 0.1075; *R*^2^ = 0.0014), indicating that the *DUG2* locus is more conducive to transcriptional activation across a longer chromosomal distance, validating our hypothesis.

### The differences seen in transcriptional activation at the integration site alter the growth phenotype and the magnitude of disruption to neighboring genes.

**FIG 4:**
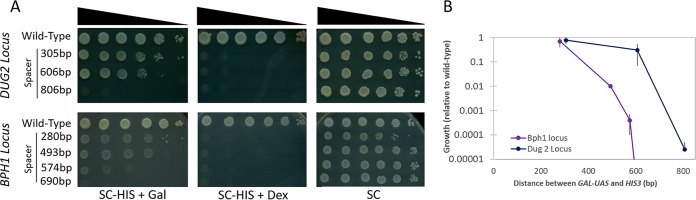
The distance of transcriptional regulation at a locus alters the growth phenotype. (A) Representative spotting assays comparing the differences in strain growth at the *DUG2* locus (top) and the *BPH1* locus (bottom). Each strain was grown for 72 h to saturation and washed with water, and 10-fold serial dilutions were spotted on the indicated plates. The cultures on the plates were grown for 72 h and imaged. Strain growth was scored as relative to wild-type growth across multiple replicates. (B) Multiple replicates from panel A were averaged and plotted as a function of the size of the spacer insert. The relative growth of each strain is compared to growth of the wild type, and error bars depict standard errors.

In order to observe whether the difference in the activation of transcription at each locus manifests as a difference in the phenotype, we utilized a spotting assay to measure the growth of each strain upon construct activation (Fig. 4A). Tenfold serial dilutions of yeast cells were spotted on agar plates activating the reporter (SC-HIS+Galactose), repressing the reporter (SC-HIS+Dextrose), or on nonselective medium (SC as a positive control). Growth was quantified, compared to the wild type, and plotted as a function of spacer distance (Fig. 4B). Regardless of the site of construct integration, when the spacer size was shortest, the level of growth approached wild-type levels. As the size of the spacer increases, a measurable decrease in growth is observed at both loci, with the level of growth dropping quicker at the *BPH1* locus than at the *DUG2* locus.

The EP theory postulates that transcriptional effects from *cis* regulatory sequences can affect transcription across a genomic region in a nonspecific, promiscuous manner. To test the EP theory, we utilized qRT-PCR to measure transcription of the genes flanking the sites of reporter integration. Gene expression was measured for the genes flanking the sites of integration under conditions activating transcription of the UAS_Gal_–*HIS3* construct. The relative expression for the genes flanking the *DUG2* site, *SAF1* and *MRPL27*, and the genes flanking the *BPH1* site, *RPS14A*, *SNT1*, and *ELO2* were measured (Table 1 and Table 2, respectively). At both loci, an increase in transcription is observed for the genes surrounding the integration site upon *HIS3* activation. The magnitude of disruption at the *DUG2* locus was larger than the disruption observed at the *BPH1* locus and varies with insert size; however, this did not appear to be a linear relationship.

**TABLE 1:** Transcription levels of the genes flanking reporter integration at the *DUG2* locus on chromosome II

Gene	Transcription level with the following spacer size (bp)^*a*^:					
	305		606		806	
	RE	SE	RE	SE	RE	SE
*SAF1*	12.325	1.816	7.192	0.668	15.439	3.568
*MRPL27*	7.300	4.880	3.831	2.872	1.116	0.275

a Transcription levels of genes flanking reporter integration at the DUG2 locus. RE, relative enrichment (compared to *ACT1*); SE, standard error.

**TABLE 2:** Transcription levels of the genes flanking reporter integration at the *BPH1* locus on chromosome III

Gene	Transcription level with the following spacer size (bp)^*a*^:							
	280		493		574		690	
	RE	SE	RE	SE	RE	SE	RE	SE
*RPS14A*	1.643	0.738	1.462	0.201	4.140	2.801	0.887	0.446
*SNT1*	0.999	0.001	9.057	2.120	11.521	2.963	2.637	1.349
*ELO2*	0.429	0.097	1.893	0.176	4.324	7.948	5.814	3.247

a Transcription levels of genes flanking reporter integration at the BPH1 locus. RE, relative enrichment (compared to *ACT1*); SE, standard error.

It should be noted that the construct integrated at the *DUG2* locus contains two selectable markers used in strain construction, *TRP1* and *NAT^R^*, while the construct integrated at the *BPH1* locus contains the *KAN^R^* marker within the UAS_Gal_–*HIS3* reporter (Fig. 1). Though the selectable markers utilized differ at each locus, their expression is constitutive and would not change under the conditions that activate *HIS3* transcription (galactose addition to the media), thus allowing us to monitor the specific effects of *HIS3* activation.

### Functionally related genes cluster together at genomic loci more conducive to coregulation at a distance.

With the observation that the extent and magnitude of transcriptional regulation vary based on both proximity to the promoter and by locus, we tested these factors as a potential mechanism underlying adjacent gene coregulation. We employed a bioinformatic approach to determine the relationship between transcriptional coregulation as a function of distance for four coregulated gene families exhibiting a nonrandom genomic distribution: the nitrogen metabolism (NM) genes, the ribosomal protein (RP) genes, the toxin response (TR) genes, and the heat shock protein (HS) genes. These families were selected due to their variation in size. These families vary in size from 18 genes (HS), 27 genes (TR), 86 genes (NM), to 129 genes (RP). Additionally, they exhibit reciprocal expression patterns upon induction of the environmental stress response (ESR) in yeast—the NM and RP genes are downregulated, while the TR and HS genes are upregulated (30). When possible, we extracted gene expression profiles for each family member and for the 10 flanking genes (five genes in each direction). Several genes neighbored telomeres, notably members of the TR gene family, and in those cases, our analysis stopped at the last protein-coding gene on the chromosome prior to the telomere. Our analysis focuses on gene expression time courses resulting in the induction of the environmental stress response, including a heat shock time course, DNA damage response time course, oxidative stress time course, e nitrogen depletion time course, and carbon source switching (28).

**FIG 5:**
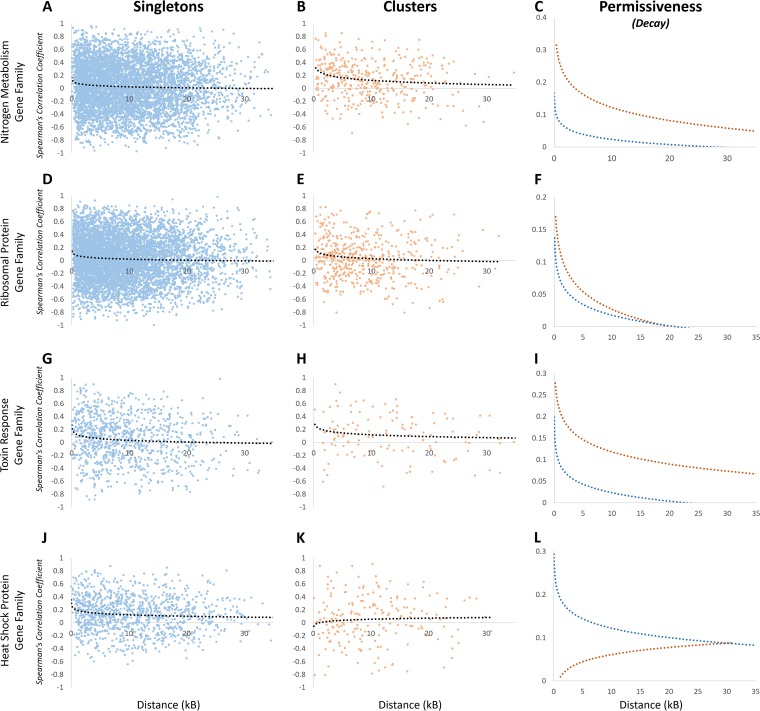
Comparison of the transcriptional coregulation across genomic regions for the singletons versus the clustered members within functionally related gene families. (A to L) Spearman’s correlation coefficient was determined with a 10-gene window for every pairwise combination for the nitrogen metabolism gene family singletons (A) and clusters (B), the ribosomal protein gene family singletons (D) and clusters (E), the toxin response gene family singletons (G) and clusters (H), and the heat shock protein gene family singletons (J) and clusters (K). The decay plots are shown with dotted black lines on each graph, and they are overlaid for comparison (C, F, I, and L).

The Spearman’s correlation coefficient was calculated for every pairwise combination of genes at each locus for every family member in each gene set. The SCC was plotted as a function of distance, separating the genes found in functional clusters from the family members found in isolation (the singletons). While significant variability in the SCC measured across the neighborhoods exists, when an exponentially decaying function was applied to the data, the singleton members of all four gene families revealed a positive correlation that decays as a function of distance (Fig. 5A, D, G, and J). For the gene members found as functional clusters throughout the genome, three gene families had a positive SCC: the NM (Fig. 5B), RP (Fig. 5E), and TR (Fig. 5H) genes. The HS genes found as clusters had a negative, anticorrelated SCC that decayed as a function of distance (Fig. 5K).

To directly compare transcriptional activation at a distance for the singleton members to the functionally clustered members of each gene family, the decay curves were extracted and overlaid onto a single plot for clarity. In three gene families, the functionally clustered members are found in genomic locations more conducive to transcriptional activation at a distance than their singleton counterparts—the NM (Fig. 5C), RP (Fig. 5F), and TR (Fig. 5I) genes. Within each of these families, the transcriptional coregulation was greater and extended over a broader genomic distance for the genes found in clusters compared to their singleton counterparts. The HS gene family exhibited the opposite relationship; the unpaired, singleton HS genes localized to more conducive genomic loci than the HS paired genes (Fig. 5L).

### Chromatin remodeling is necessary for adjacent gene coregulation.

Chromatin maintenance provides a desirable model for the coregulation of functionally related, clustered genes. A bioinformatic approach was used to identify putative chromatin remodelers required for coregulation of the clustered genes. This approach took advantage of the large-scale gene deletion study characterizing the differences in gene expression upon deletion of 165 nonessential chromatin remodeling genes (31). Gene expression data were extracted for the members of the NM, RP, TR, and HS gene families, with the extent of gene disruption determined for the clustered members of each family compared to the singleton members. The significance of disruption was calculated by a hypergeometric probability density function and focused on remodeling complexes that disrupt the clusters preferentially compared to the rest of the members within each gene family (24). Thus, our analysis looked for chromatin remodeler deletion mutants that resulted in the overrepresentation of clustered genes losing their transcriptional coregulation relative to the rest of the genes within the set. This approach would eliminate the identification of chromatin remodelers that regulate the transcription of an entire gene family and allow for the identification of those remodelers that are specific to the coregulation of clustered family members. Our analysis identified a set of chromatin remodelers that preferentially uncouple transcription of the functionally clustered genes within each family, using a threshold of *P* < 0.05 as our cutoff for transcriptional deviation from wild-type levels of expression (Table 3). The coregulation of the clustered members in the nitrogen metabolism gene family is uncoupled from the rest of the set without the SAGA complex (*hfi1* and *spt20* mutants), the Set3 histone deacetylase complex (*set3* mutant), and the Swi/Snf complex (*snf12* mutant). Likewise, the ribosomal protein gene family clusters require the activity of the NuA3 histone acetyltransferase complex (*yng1* and *sas3* mutants), the Compass complex (*sdc1* mutant), and the SAGA/SLIK complexes (*ngg1* mutant). The toxin response gene family requires the RSC complex (*rsc2* mutant), and the heat shock protein gene family requires the HIR complex (*hpc2* mutant). Altogether, our analysis identifies a role for chromatin remodeling complexes necessary to coordinate the transcription of functionally related gene clusters and underlie adjacent gene coregulation.

**TABLE 3:** Genes coding for chromatin remodelers that significantly disrupt the transcription of functional clusters relative to the unpaired members within a gene family

Family and gene	*P* value
Nitrogen metabolism gene family	
*hfi1*	0.0003
*spt20*	0.0011
*set3*	0.0142
*snf12*	0.0206
*not4*	0.0252
*bre1*	0.0268
*set2*	0.0288
*ies1*	0.0303
*cac2*	0.0303
*ssn6*	0.0388
*sif2*	0.0418
*ies5*	0.0424
*hos2*	0.0482
*sum1*	0.0482

Ribosomal protein gene family	
*yng1*	<0.0001
*rtt109*	0.0120
*med16*	0.0221
*sas3*	0.0257
*sdc1*	0.0310
*ngg1*	0.0349

Toxin response gene family	
*rsc2*	0.0433

Heat shock protein gene family	
*hpc2*	0.0245

## DISCUSSION

### The limit to transcriptional activation at a distance varies by genomic locus.

The work presented in this article expands the growing body of evidence documenting the interconnectedness of gene expression based on spatial positioning. Previous studies have characterized the impact of the position effects seen with the integration of a reporter construct in Saccharomyces cerevisiae; these effects result in large differences in expression (up to a 13-fold difference) and can disrupt the expression of the adjacent gene (the neighboring gene effect [NGE]) at a significant number of loci (approximately 7 to 15% of the genome). Transcriptional disruption by the NGE led to erroneous attribution to gene function (32–34). The distance that regulatory elements can function has similarly been characterized, depending on both proximity to a gene (typically 450 bp in yeast) and the activity of the Mediator complex (26, 35). In the present work, we verify and extend these studies, demonstrating that even seemingly subtle differences across genomic loci can profoundly impact the extent of transcriptional activation at a distance.

The understanding of how a promoter regulates transcription on a cellular level has evolved, with recent findings characterizing the EP effect—that *cis* regulatory regions exert their effects over broad distances which can result in alterations in transcriptional regulation throughout a genomic neighborhood (16). The EP effect has been theorized to affect transcription in widely divergent species, from budding yeast up through humans; however, it was empirically tested only in Caenorhabditis elegans. Our study builds upon this work and adds to the growing body of evidence that the EP effect may be ubiquitous in eukaryotes. Our work also demonstrates the limiting effects that a genomic locus imposes on this phenomenon, showing that the differences between genomic loci can manifest as phenotypic differences and can result in differences in the transcriptional disruption that vary with genomic neighborhood.

### Adjacent gene coregulation is mediated, in part, by the clustering of genes at loci conducive to transcriptional activation at a distance.

The RP and RRB regulons both exhibit a nonrandom genomic distribution, occurring as functional pairings (21, 36). Functional dissection of one pair of RRB genes, the *MPP10-MRX12* gene pair, revealed a dependency on physical adjacency for their coordinated transcription with the rest of the RRB regulon. The physical separation of *MRX12* from *MPP10* results in the uncoupled expression of *MRX12* (but not *MPP10*) from the rest of the RRB gene family (23). The coregulation of this gene pair depends on two shared promoter motifs found upstream of *MPP10*, which recruits transcription factors to the locus, and results in changes to the chromatin to the region (23). This phenomenon is called adjacent gene coregulation, and follow-up work revealed the dependency of chromatin remodelers for its maintenance at this locus (24). It was subsequently observed that roughly 25% of gene families exhibit a nonrandom positioning throughout the genome of S. cerevisiae, predominantly found as functionally related clusters (25).

One challenge to understanding the functional clustering causing adjacent gene coregulation is understanding what mechanisms underlie this process. The gene families found as functional clusters are diverse and varied, belonging to many different ontology classes (25). Each gene family is characterized by its own *cis* and *trans* factors, with many families exhibiting a multitude of additional layers. Here we propose the process that ultimately gives rise to functional clusters may be passive in nature—the result of chance genomic rearrangements over time resulting in two functionally related genes clustering together into a genomic region that is more conducive for coexpression. This would allow for long-range transcriptional control to mediate transcription of the members of a given regulon, effectively streamlining the genome (37). Additional support exists for this interpretation: genes that are physically clustered together are not conserved over evolutionary distance; however, the absolute numbers of clustered genes are comparable (22, 23, 25). For example, Candida albicans and Schizosaccharomyces pombe each exhibit the same levels as pairing as S. cerevisiae for both the RP and RRB genes, although the genes clustered differ across these species (23).

The genic arrangement into clusters can also play a role in regulating mutually exclusive expression at a locus. This is evident in our analysis of the heat shock protein gene family. The expression of one gene interferes with the expression of the neighboring gene; they exhibit a reciprocal expression pattern and are anticorrelated. Such an arrangement has been observed and extensively characterized at the *SER3* locus, which is regulated in a mutually exclusive manner by the adjacent noncoding gene *SRG1* (38, 39). Transcription of *SRG1* inhibits the transcription of *SER3*, with the intergenic repression mediated by nucleosome assembly (40).

### Role of chromatin remodeling in adjacent gene coregulation.

The EP theory demonstrates that *cis* regulatory sequences influence transcription throughout a genomic region (16). Changes to chromatin structure at a locus can modulate transcription throughout a region; we explored this possibility in coordinating the transcription of clustered genes. By taking advantage of the comprehensive screen monitoring gene expression changes after deletion of the nonessential genes comprising components of chromatin remodeling complexes, we identified putative regulators of adjacent gene coregulation (31). By focusing our analysis on the remodelers that disproportionately affect functional clusters compared to the singleton members in a gene family, our analysis excluded complexes that are specific regulators of each gene family. Our results implicate several chromatin remodeling complexes that are known regulators of transcription at a distance (Mediator) and coregulation at the *SRG1*-*SER3* locus (SAGA and the Swi/Snf complexes) (35, 39). We also identify additional regulatory complexes not previously identified in this process, including the NuA3 complex, RSC complex, and HIR complex. Our analysis identified several components from the same remodeling complexes, although there were several examples where only a single component was identified from a complex with multiple components. This observation requires further follow-up analysis and verification and are beyond the scope of this work.

### Conclusion.

The clustering of coregulated genes throughout the genome allows for greater transcriptional coordination by allowing for regulatory mechanisms to reinforce each other throughout a locus. The extension of the EP theory presented here extends the understanding of the wide-spread nature of this phenomenon and helps to complete the picture of how correlated domains of expression arise to streamline transcriptional coregulation. Such a mechanism extends throughout eukaryotic species of diverse lineages and complexity. Our work provides insight into the interconnectedness of spatial positioning in gene expression, with potential applications to the understanding of spatial positioning in humans and disease (41–43).

## MATERIALS AND METHODS

### Yeast strains and growth conditions.

The complete list of S. cerevisiae strains utilized in this study and relevant genes can be found in Table 4. For RNA extraction and gene expression analysis, strains of yeast were grown overnight at 30°C in YPAD (1% yeast extract, 2% peptone, 40 mg adenine, 2% dextrose [all per liter]) medium between early and mid-log phase (optical density at 600 nm [OD_600_], 0.40 to 0.90). Cultures of yeast were pelleted, washed once with ddH_2_O, and split prior to resuspension in selective medium (SC-HIS+galactose) and nonselective medium (SC-HIS+dextrose) for 2 or 3 doublings (to mid-log-phase growth) prior to RNA extraction. For the spotting assay, strains of yeast were grown at 30°C in YPAD medium to post-log phase, washed with ddH_2_O, and then serially diluted 10-fold. Three microliters of each dilution was then spotted onto selective plates (SC-HIS+galactose), nonselective plates (SC-HIS+dextrose), and control (SC) plates. Yeast cultures on the plates were grown for 72 h prior to imaging and analysis.

**TABLE 4:** Complete list of Saccharomyces cerevisiae strains used in this study and relevant genotypes

Strain	Spacer (bp)	Relevant genotype
YJA1508		*MAT****a*** *ura3-52* (wild type) (no spacer)
YJA1509	280	*MATα his3Δ200 lys2Δ128 leu2Δ0 ura3Δ0 trpΔ63 bph1Δ*::*kanMX-UASGAL280-HIS3*
YJA1511	493	*MATα his3Δ200 lys2Δ128 leu2Δ0 ura3Δ0 trpΔ63 bph1Δ*::*kanMX-UASGAL493-HIS3*
YJA1512	574	*MATα his3Δ200 lys2Δ128 leu2Δ0 ura3Δ0 trpΔ63 bph1Δ*::*kanMX-UASGAL574-HIS3*
YJA1513	690	*MATα his3Δ200 lys2Δ128 leu2Δ0 ura3Δ0 trpΔ63 bph1Δ*::*kanMX-UASGAL690-HIS3*
YJA1515	305	*MATα his3Δ200 leu2Δ0 ura3Δ0 trp1Δ63 dug2Δ*::*TRP1-UASGAL305-HIS3-natMX*
YJA1516	606	*MATα his3Δ200 leu2Δ0 ura3Δ0 trp1Δ63 dug2Δ*::*TRP1-UASGAL606-HIS3-natMX*
YJA1517	806	*MATα his3Δ200 leu2Δ0 ura3Δ0 trp1Δ63 dug2Δ*::*TRP1-UASGAL806-HIS3-natMX*

### RNA extraction and gene expression analysis.

Portions (15 ml) of yeast cultures were pelleted at 3,000 (3K) rpm for 3 min at room temperature and washed once with ddH_2_O. RNA was extracted using the ZR Fungal/Bacterial RNA MiniPrep kit (Zymo Research, CA) with the modifications outlined as follows. Pellets of yeast cells were resuspended in 800 µl of RNA lysis buffer and transferred to a ZR Bashing Beads lysis tube, and cells were lysed with mechanical shearing by vortex (30-s vortex followed by 60-s incubation on ice for a total of eight cycles). DNase I digestion was performed in a column utilizing the RNase-free DNase I kit (Life Technologies Corporation, CA), and RNA was eluted into 25 μl of RNase-free water. The efficacy of DNase I treatment was verified by endpoint PCR, targeting an *ACT1* amplicon.

cDNA was then synthesized using the TaqMan reverse transcription kit (Life Technologies Corporation, CA). DNase-treated RNA (500 to 1,000 ng) was utilized per reaction mixture using oligo(dT) primers, and cDNA was synthesized using a thermocycler according to the manufacturer’s instructions. Newly synthesized cDNA was verified by amplification using endpoint PCR targeting the *ACT1* amplicon. Gene expression was then measured by qPCR utilizing PowerUp Sybr Green Master Mix (Life Technologies Corporation, CA) and a final reaction mixture volume of 25 µl per reaction. Triplicate RNA reactions were obtained (biological replicates), and at least three technical replicates were performed per sample. Reactions that did not amplify or were nonspecific (e.g., more than a single peak observed on a melt curve analysis) were discarded from our analysis. Relative expression and fold enrichment were determined using 2^ΔΔCT^ method with *ACT1* as a reference (44). The complete list of PCR primers and their sequences utilized for qPCR analysis can be found in Table 5.

**TABLE 5:** Complete sequences of PCR primers utilized in this study

Primer	Target	Sequence (5′ – 3′)
prJA0047	*HIS3* FP	CAGAAGCAGTAGCAGAACAG
prJA0048	*HIS3* RP	ATGGTCGTCTATGTGTAAGTC
prJA0051	*RPS14A* FP	AGAGTTACTGGTGGTATGAAG
prJA0052	*RPS14A* RP	CTGGAGTCTTGGTTCTAGTAC
prJA0053	*ELO2* FP	CTGGTGGAAGGAATGGGTTAC
prJA0054	*ELO2* RP	AGTTGTTGAACCCACACAGTC
prJA0055	*MRPL27* FP	TAAAAGAGTGCCGTTGACCAC
prJA0056	*MRPL27* RP	TGGGGTAACATAGGTTCTGAC
prJA0057	*SAF1* FP	TCCCAGTGGTTCACAGCATGA
prJA0058	*SAF1* RP	CCGTGGTATTGACAGTACTC
prJA0063	*SNT1* FP	AGGGGTGTATTTTCCCATTAC
prJA0064	*SNT1* RP	GTGCGTAAAAAGGATACTCTG

### Determination of transcriptional coregulation at a distance and calculation of the Spearman’s correlation coefficient.

The Spearman’s correlation coefficient was computed as described previously utilizing the formula:

$$\rho =\frac{\mathrm{cov}(g_{1},g_{2})}{\sigma_{g_{1}}\times \sigma_{g_{2}}}$$

where *g*_1_ and *g*_2_ are the corresponding genes for comparison, *cov* is their covariance, and σ is their standard deviation (SD) (16). Gene expression profiles were obtained from previously published microarray data sets and analyzed. Data were extracted for the following conditions for analysis: cells cycling through the cell cycle;, chemostat growth limiting for carbon, nitrogen, phosphorus, or sulfur; heat shock induction by shifting cultures growing at 30°C to 37°C; oxidative stress induced by the addition of 0.3mM hydrogen peroxide, DNA damage induced by the addition of 0.03% MMS, nitrogen starvation induced by growth in SD media, and carbon source shifted from glucose to glycerol. Prior to the analysis, duplicate data points were averaged together to generate a single time series for each gene and then normalized to *TDH3* expression to allow comparisons across different microarray data sets (27–29). The SCC was calculated for every pairwise combination of genes within a 10-gene window surrounding each locus or until the end of the chromosome was reached. Data were plotted and fit to a decay curve in Excel for analysis.

### Identification of chromatin remodelers that disrupt adjacent gene coregulation.

The data set from the chromatin interaction deletion study was analyzed to identify mutants that preferentially disrupted transcription of the functionally clustered genes relative to that of the unpaired members of the same set (threshold of *P* < 0.05) in the NM, RP, TR, and HS gene sets. This threshold of transcriptional disruption represents the deviation in transcription measured in a mutant strain compared to an isogenic, wild-type strain of yeast. The significance was subsequently determined utilizing a hypergeometric probability density function:

$$P=1-\overset{}{\underset{k}{\sum }}\frac{\left(\frac{K}{k})(\frac{N-K}{n-k}\right)}{\left(\frac{N}{n}\right)}$$

where *P* is the probability, *K* is the total number of genes disrupted, *k* is the number of genes in the subset disrupted, *n* is the number of genes in the subset, and *N* is the total number of genes with measured *P* values in the microarray data set as previously described (24, 31).
